# Draft genome sequence of *Aspergillus oryzae* (Ahlburg) Cohn ATCC 16868

**DOI:** 10.1128/mra.01100-24

**Published:** 2025-02-24

**Authors:** Berenice Talamantes Becerra, Mya Myintzu Hlaing, Catherine M. McAuley, Natalie A. Twine, Denis C. Bauer, Netsanet Shiferaw Terefe

**Affiliations:** 1 Australian e-Health Research Centre, Commonwealth Scientific and Industrial Research Organisation, Sydney, New South Wales, Australia; 2 A&F, Commonwealth Scientific and Industrial Research Organisation, Werribee, Victoria, Australia; 3 Department of Applied BioSciences, Macquarie University 7788 , Sydney, New South Wales, Australia; 4 Department of Biomedical Science, Macquarie University 7788 , Sydney, New South Wales, Australia; University of California Riverside, Riverside, California, USA

**Keywords:** genome assembly, *Aspergillus oryzae*

## Abstract

The draft genome sequence of *Aspergillus oryzae* ATCC 16868 is presented here. *A. oryzae* produces carbohydrase and proteolytic enzymes and is used in the production of fermented food and feed. Genomic data have been utilized to investigate allergenicity and virulence characteristics, contributing to its safety evaluation in food production systems.

## ANNOUNCEMENT

*Aspergillus oryzae* (Ahlburg) Cohn ATCC 16868, NRRL 451, C. Thom 4235.15, was obtained in lyophilized form from ATCC and cultured according to the manufacturer’s instructions (1). The culture was maintained prior to DNA isolation, and it is preserved as part of the CSIRO Food Fungal Culture Collection. *A. oryzae* produces a wide range of amylolytic and proteolytic enzymes, as well as enzymes that degrade plant-derived polysaccharides (2). The degradation of non-protein components, such as fibers and complex carbohydrates in biomass, enhances the availability of nutrients, particularly proteins and amino acids, already present in the substrate. In aquaculture, the use of fermented plant-based ingredients, such as canola meal or grain by-products, represents an economical and environmentally friendly alternative for feeding aquatic species (3). Fermentation-induced improvement in nutritional content reduces the need for additives, making the process more sustainable and cost-effective. The genome sequencing of *A. oryzae* enables the *in silico* identification of virulence factors and allergenic potential in fungi used in fermentation, which is critical for safety assessments.

Genomic DNA for *A. oryzae* ATCC 16868 was prepared as follows. DNA was isolated from processed cell pellets using the Quick-DNA Fungal/Bacterial Miniprep Kit (Zymo Research) following the procedures described by the manufacturer. The DNA concentration was measured using a Qubit Fluorometer and Qubit DNA HS assay Kits (Life Technologies, Australia). The DNA quality was evaluated with an Agilent 2200 TapeStation system using the Agilent DNA ScreenTape assay. The extracted DNA was stored at −20°C. The library preparation was carried out using the Nextera XT DNA Library Preparation Kit, following the guidelines outlined in the Nextera XT DNA Library Prep Kit Reference Guide (15031942 version 03). Sequencing was performed on the NextSeq 550 system (Illumina, San Diego, CA, USA) using a 2 × 150 bp paired-end configuration. Illumina short reads were processed for quality control and trimming adapters using Trimgalore version 0.6.10 (4) with default parameters. We utilized SPAdes version 3.15.5 (5) for *de novo* assembly of Illumina PE data, using the --isolate option to optimize assembly for isolates and k-mer lengths of 21, 33, 55, 77, 99, and 127. Genome assembly quality and summary statistics were generated using metaQuast version 5.2.0 (6) with default parameters. The completeness of the assembled genomes was performed using BUSCO version 5.2.2 (7) with the fungi_odb10 database and default settings. A total of 10,194,765 pairs of paired-end reads were sequenced, and 10,191,532 remained after filtering and trimming. After performing a QC and trimming adapters, the assembly resulted in 724 contigs, with a total length of 36,838,118 bp (*N*_50_ 131,073 bp; *N*_90_ 38,039 bp), a G + C content of 48.3%, and genome completeness of 98.7% (Table 1).

**TABLE 1:** Genome sequencing statistics

Parameter	Value
Illumina sequencing	
Number of reads (sum of R1 and R2)	20,389,530
Total bases (bp)	3,078,819,030
Genome assembly	
Total length	36,838,118
Total contigs	724
Maximum sequence length	627,805
*N*_50_	130.9 kb
*N*_90_	38,039 bp
GC content (%)	48.5
Average depth of genome coverage	10×
Genome assessment	
Complete BUSCOs	98.7%
Complete and single-copy BUSCOs	98.2%
Complete and duplicated BUSCOs	0.5%
Fragmented BUSCOs	0.5%

## Data Availability

This Whole Genome Shotgun project has been deposited at DDBJ/ENA/GenBank under the accession JBHVOD000000000. The version described in this paper is version JBHVOD010000000. The Sequence Read Archive (SRA) accession number is SRR30858638. Both the WGS and SRA data are associated with the project PRJNA1164934.
